# Supplementary material for: Identification of responsible sequences which mutations cause maternal H19-ICR hypermethylation with Beckwith–Wiedemann syndrome-like overgrowth
Source: Commun Biol. 2024 Dec 2;7:1605. doi: 10.1038/s42003-024-07323-x (PMC11612015; doi:10.1038/s42003-024-07323-x)
Supplement: Supplementary file 2 — Supplemental information [file 42003_2024_7323_MOESM2_ESM.pdf]

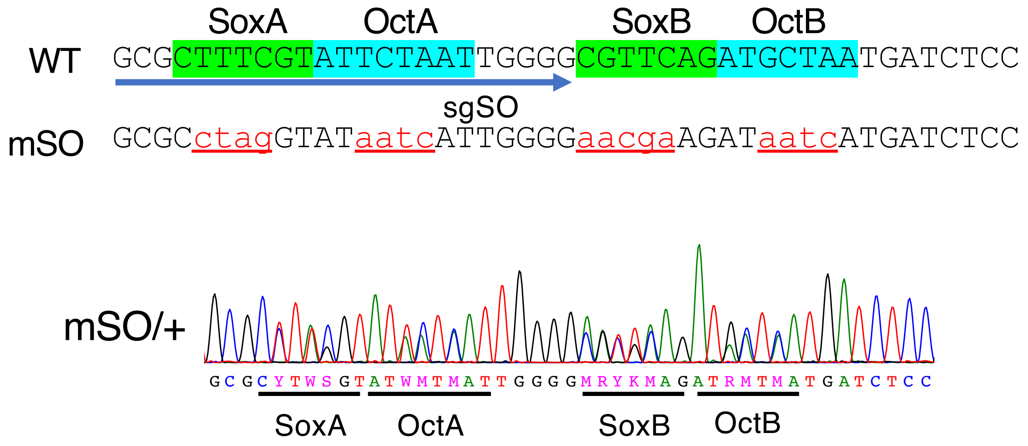

Supplementary Figure 1.

(top) Nucleotide sequences of SOBS. Wild-type (WT) and mutant (*H19*-ICR^{mSO}; mSO) alleles are shown. SOX- (SoxA/B) and OCT-binding sites (OctA/B) are highlighted in green and blue, respectively. Blue arrow indicates the target sequence of sgRNA and its direction. Mutated sequences of mSO are shown in red underlined letters. (bottom) Representative electropherogram of a heterozygous mutant in the F1 generation. Black underlines indicate SoxA/B and OctA/B.

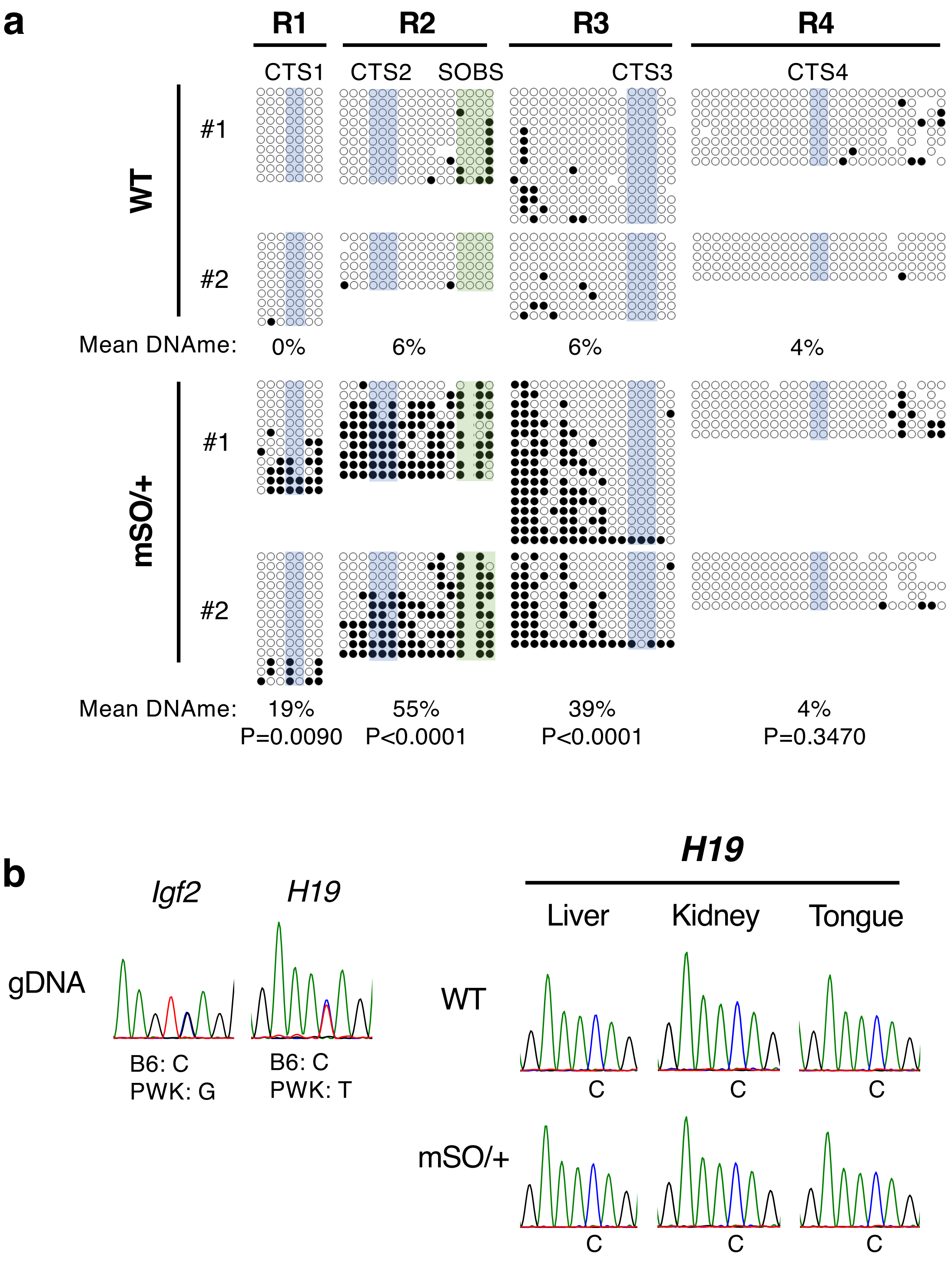

Supplementary Figure 2.
(a) Full results of the methylation status of the maternal allele in WT and *H19*-ICR$^{mSO/+}$ neonates. Percentages indicate mean DNA methylation levels of two replicates. *P*-values show statistical differences between WT and *H19*-ICR$^{mSO/+}$ tissues (Mann-Whiteney U test). (b) Allelic expression of *H19* in WT and *H19*-ICR$^{mSO/+}$ tissues. Representative electropherograms of the products amplified from genomic DNA (gDNA) and cDNA are shown.

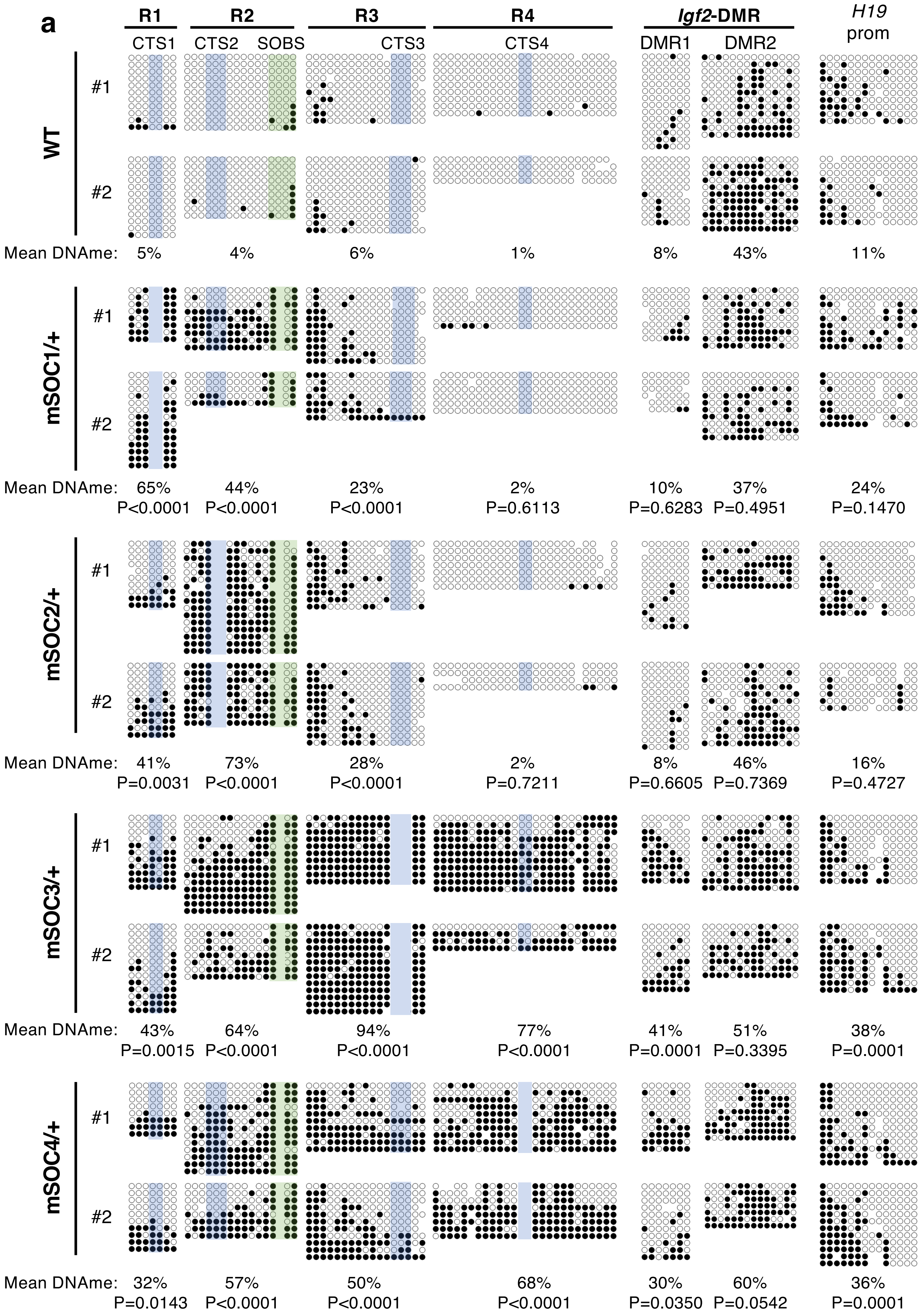
a
R1
R2
R3
R4
Igf2-DMR
H19 prom
CTS1
CTS2
SOBS
CTS3
CTS4
DMR1
DMR2
WT
#1
#2
Mean DNAme: 5% 4% 6% 1% 8% 43% 11%
mSOC1/+
Mean DNAme: 65% 44% 23% 2% 10% 37% 24%
P<0.0001 P<0.0001 P<0.0001 P=0.6113 P=0.6283 P=0.4951 P=0.1470
mSOC2/+
Mean DNAme: 41% 73% 28% 2% 8% 46% 16%
P=0.0031 P<0.0001 P<0.0001 P=0.7211 P=0.6605 P=0.7369 P=0.4727
mSOC3/+
Mean DNAme: 43% 64% 94% 77% 41% 51% 38%
P=0.0015 P<0.0001 P<0.0001 P<0.0001 P=0.0001 P=0.3395 P=0.0001
mSOC4/+
Mean DNAme: 32% 57% 50% 68% 30% 60% 36%
P=0.0143 P<0.0001 P<0.0001 P<0.0001 P=0.0350 P=0.0542 P=0.0001

(continued)

b

	mSOC1			mSOC2			mSOC3			mSOC4		
	DNAme #1 (%)	DNAme #2 (%)	p-value #1 vs #2	DNAme #1 (%)	DNAme #2 (%)	p-value #1 vs #2	DNAme #1 (%)	DNAme #2 (%)	p-value #1 vs #2	DNAme #1 (%)	DNAme #2 (%)	p-value #1 vs #2
R1	75	59	0.3639	27	52	0.1723	50	38	0.4206	38	34	0.7994
R2	30	55	0.1918	79	70	0.1100	56	68	0.2947	57	57	0.9825
R3	23	23	0.7882	24	25	0.8402	92	95	0.5009	59	40	0.0975
R4	0	3	0.4615	2	3	1.0000	72	60	0.2967	64	69	0.4209
Igf2-DMR1	5	15	0.3706	12	9	0.5734	45	41	0.8651	43	21	0.1995
Igf2-DMR2	42	32	0.3626	48	44	0.7273	50	49	0.8545	55	65	0.7352
H19prom	29	45	0.2494	20	11	0.4070	27	43	0.1030	31	38	0.2724

c

	mSOC1		mSOC2		mSOC3		mSOC4	
	p-value #1 vs WT	p-value #2 vs WT	p-value #1 vs WT	p-value #2 vs WT	p-value #1 vs WT	p-value #2 vs WT	p-value #1 vs WT	p-value #2 vs WT
R1	<0.0001	<0.0001	0.0216	0.0001	0.0004	0.0011	0.0262	0.0203
R2	<0.0001	0.0010	<0.0001	<0.0001	<0.0001	0.0002	<0.0001	0.0001
R3	0.0002	0.0038	0.0001	<0.0001	<0.0001	<0.0001	0.0001	0.0001
R4	0.6780	0.5211	1.0000	0.6941	<0.0001	0.0004	0.0002	0.0002
Igf2-DMR1	0.8256	0.2964	0.6584	0.9736	0.0007	0.0146	0.0278	0.2641
Igf2-DMR2	0.9240	0.3293	0.6973	0.8681	0.2445	0.8159	0.1841	0.0798
H19prom	0.0719	0.6486	0.8966	0.2075	0.0882	0.0016	0.1556	0.0047

Supplementary Figure 3.
(a) Full results of the methylation status of the maternal allele in WT and *H19*-ICR$^{mSOC1\text{-}4/+}$ neonates. Percentages indicate mean DNA methylation levels of two replicates. *P*-values show statistical differences between WT and *H19*-ICR$^{mSOC1\text{-}4/+}$ neonates (Mann-Whiteney U test). (b and c) Comparison of DNA methylation levels at H19-ICR and somatic DMRs. *P*-values show statistical differences between biological replicates (b) and each biological replicate versus WT (c, Mann-Whiteney U test). *P*-values less than 0.05 are indicated in red.

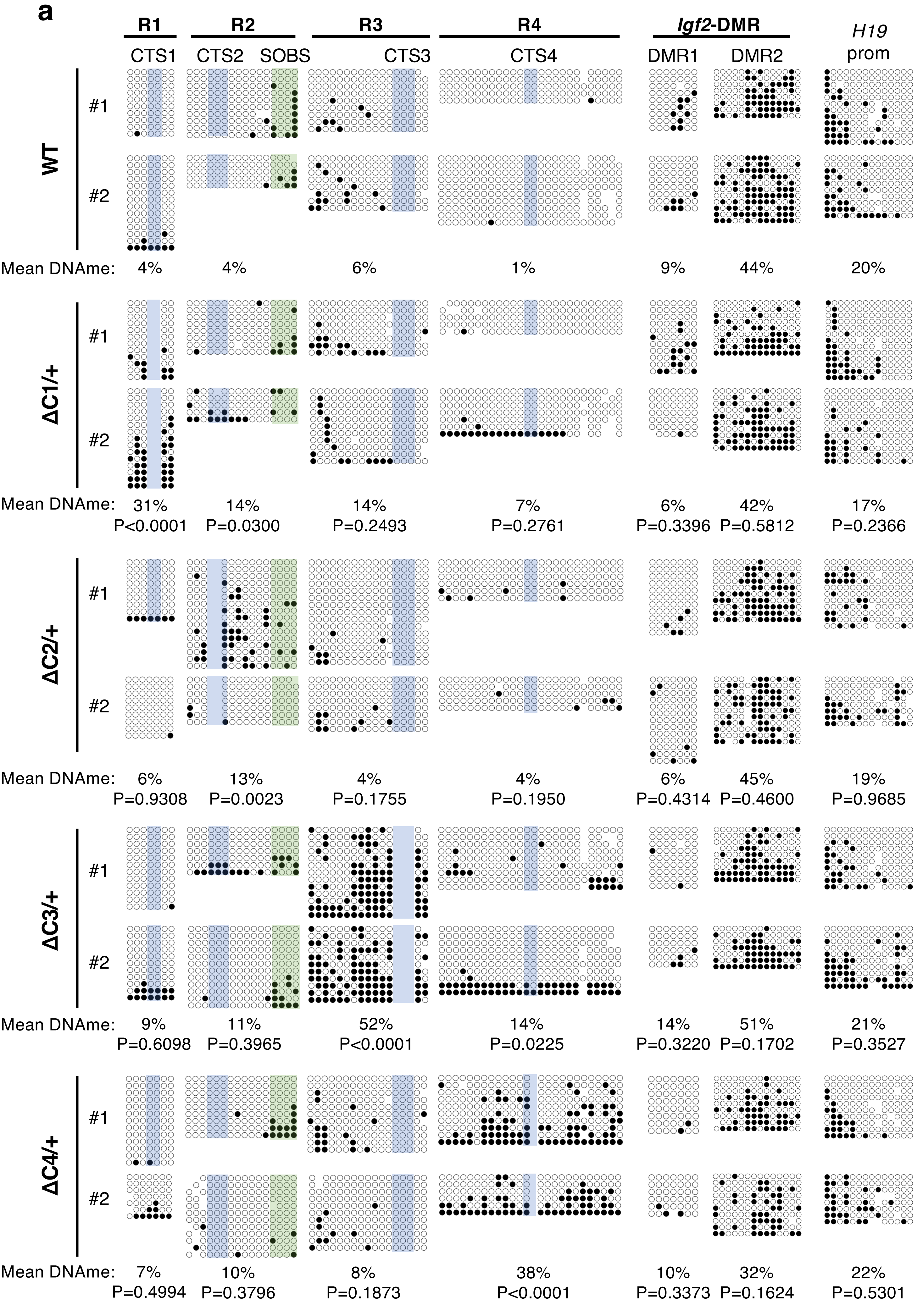
a
R1
R2
R3
R4
Igf2-DMR
H19 prom
CTS1
CTS2
SOBS
CTS3
CTS4
DMR1
DMR2
WT
#1
#2
Mean DNAme: 4% 4% 6% 1% 9% 44% 20%
ΔC1/+
Mean DNAme: 31% 14% 14% 7% 6% 42% 17%
P<0.0001 P=0.0300 P=0.2493 P=0.2761 P=0.3396 P=0.5812 P=0.2366
ΔC2/+
Mean DNAme: 6% 13% 4% 4% 6% 45% 19%
P=0.9308 P=0.0023 P=0.1755 P=0.1950 P=0.4314 P=0.4600 P=0.9685
ΔC3/+
Mean DNAme: 9% 11% 52% 14% 14% 51% 21%
P=0.6098 P=0.3965 P<0.0001 P=0.0225 P=0.3220 P=0.1702 P=0.3527
ΔC4/+
Mean DNAme: 7% 10% 8% 38% 10% 32% 22%
P=0.4994 P=0.3796 P=0.1873 P<0.0001 P=0.3373 P=0.1624 P=0.5301

(continued)

b

	ΔC1			ΔC2			ΔC3			ΔC4		
	DNAme #1 (%)	DNAme #2 (%)	p-value #1 vs #2	DNAme #1 (%)	DNAme #2 (%)	p-value #1 vs #2	DNAme #1 (%)	DNAme #2 (%)	p-value #1 vs #2	DNAme #1 (%)	DNAme #2 (%)	p-value #1 vs #2
R1	17	45	0.0397	11	2	0.0617	1	18	0.1218	2	19	0.0542
R2	17	20	0.6204	16	3	0.0084	17	8	0.7084	10	3	0.3138
R3	11	5	0.7340	3	7	0.2868	56	49	0.4477	10	5	0.0928
R4	1	12	0.3990	4	4	0.9134	9	20	0.2330	31	38	0.9354
Igf2-DMR1	18	2	0.0131	6	7	0.8221	14	3	0.1608	4	9	0.7063
Igf2-DMR2	38	47	0.3018	46	43	0.6201	44	49	0.3736	32	35	0.6577
H19prom	18	12	0.3403	16	24	0.2783	19	28	0.5580	17	30	0.0567

c

	ΔC1		ΔC2		ΔC3		ΔC4	
	p-value #1 vs WT	p-value #2 vs WT	p-value #1 vs WT	p-value #2 vs WT	p-value #1 vs WT	p-value #2 vs WT	p-value #1 vs WT	p-value #2 vs WT
R1	0.0217	0.0001	0.9418	0.8552	0.7821	0.2922	0.6011	0.0853
R2	0.6762	0.2534	0.0001	0.9293	0.4634	0.5034	0.2642	0.9667
R3	0.3857	0.2372	0.0839	0.7641	<0.0001	<0.0001	0.0960	0.8967
R4	0.8407	0.1841	0.1950	0.0532	0.0030	0.1724	0.0002	<0.0001
Igf2-DMR1	0.0869	0.2307	0.6099	0.5295	0.2070	0.8534	0.2045	0.5945
Igf2-DMR2	0.3413	0.9811	0.7215	0.3700	0.8909	0.6752	0.4151	0.1776
H19prom	0.5468	0.1734	0.5011	0.4510	0.7899	0.4059	0.5129	0.0661

Supplementary Figure 4.
Full results of the methylation status of the maternal allele in WT and *H19*-ICR$^{\Delta C1\text{-}4/+}$ neonates. Percentages indicate mean DNA methylation levels of two replicates. *P*-values show statistical differences between WT and *H19*-ICR$^{\Delta C1\text{-}4/+}$ neonates (Mann-Whiteney U test). (b and c) Comparison of DNA methylation levels at H19-ICR and somatic DMRs. *P*-values show statistical differences between biological replicates (b) and each biological replicate versus WT (c, Mann-Whiteney U test). *P*-values less than 0.05 are indicated in red.

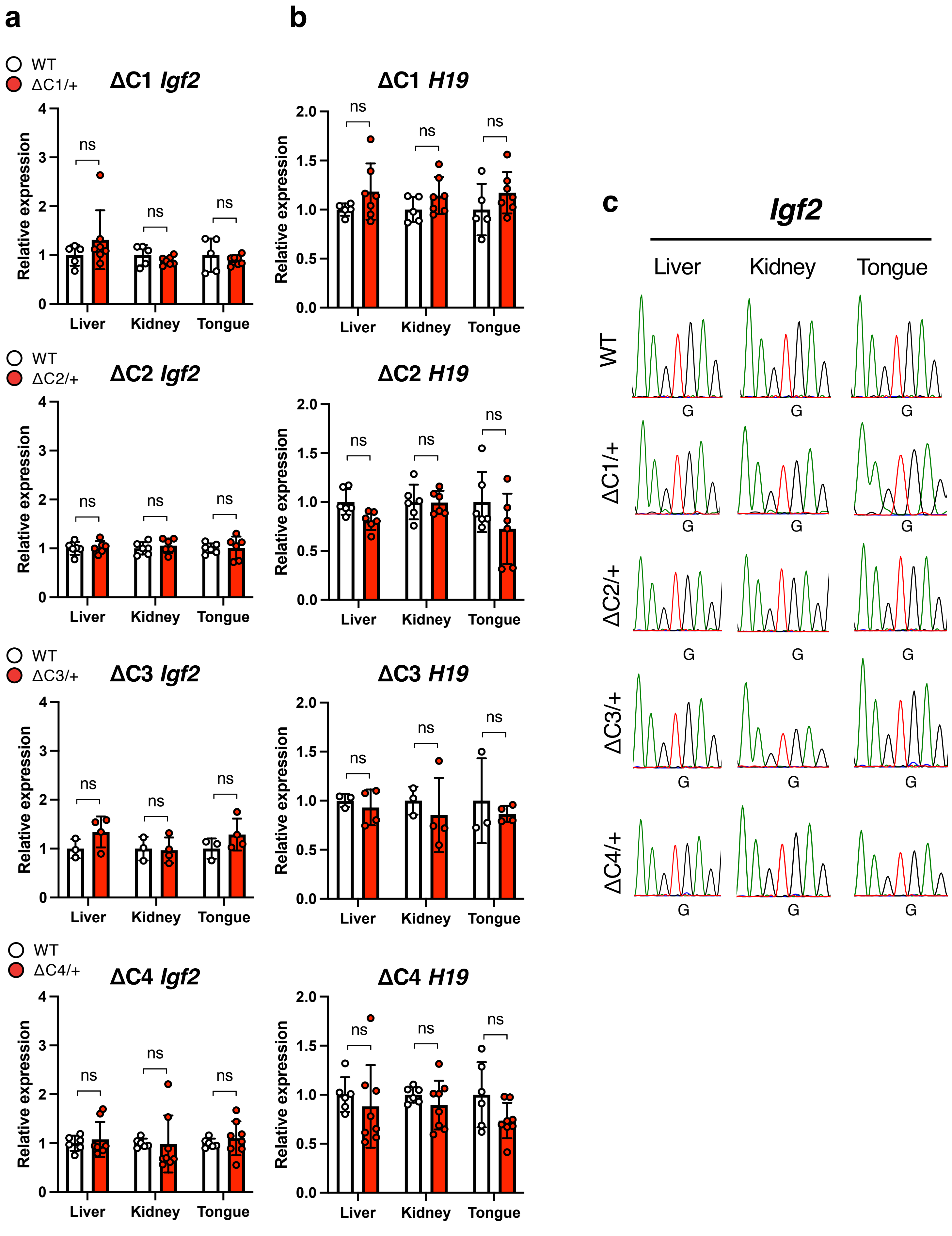

Supplementary Figure 5.

(a and b) Expression levels of *Igf2* (a) and *H19* (b) in *H19*-ICR$^{\Delta C1/+}$ (n = 8) and WT (n = 8), *H19*-ICR$^{\Delta C2/+}$ (n = 6) and WT (n = 4), *H19*-ICR$^{\Delta C3/+}$ (n = 4) and WT (n = 3), and *H19*-ICR$^{\Delta C4/+}$ (n = 6) and WT (n = 8) tissues from 2 litters. White and red bars indicate WT and mutant tissues, respectively. Error bars indicate standard deviation. ns; not significant (unpaired two-tailed *t*-test). (c) Allelic expression of *Igf2* in WT and *H19*-ICR$^{\Delta C1\text{-}4/+}$ tissues. Representative electropherograms of the RT-PCR products are shown.

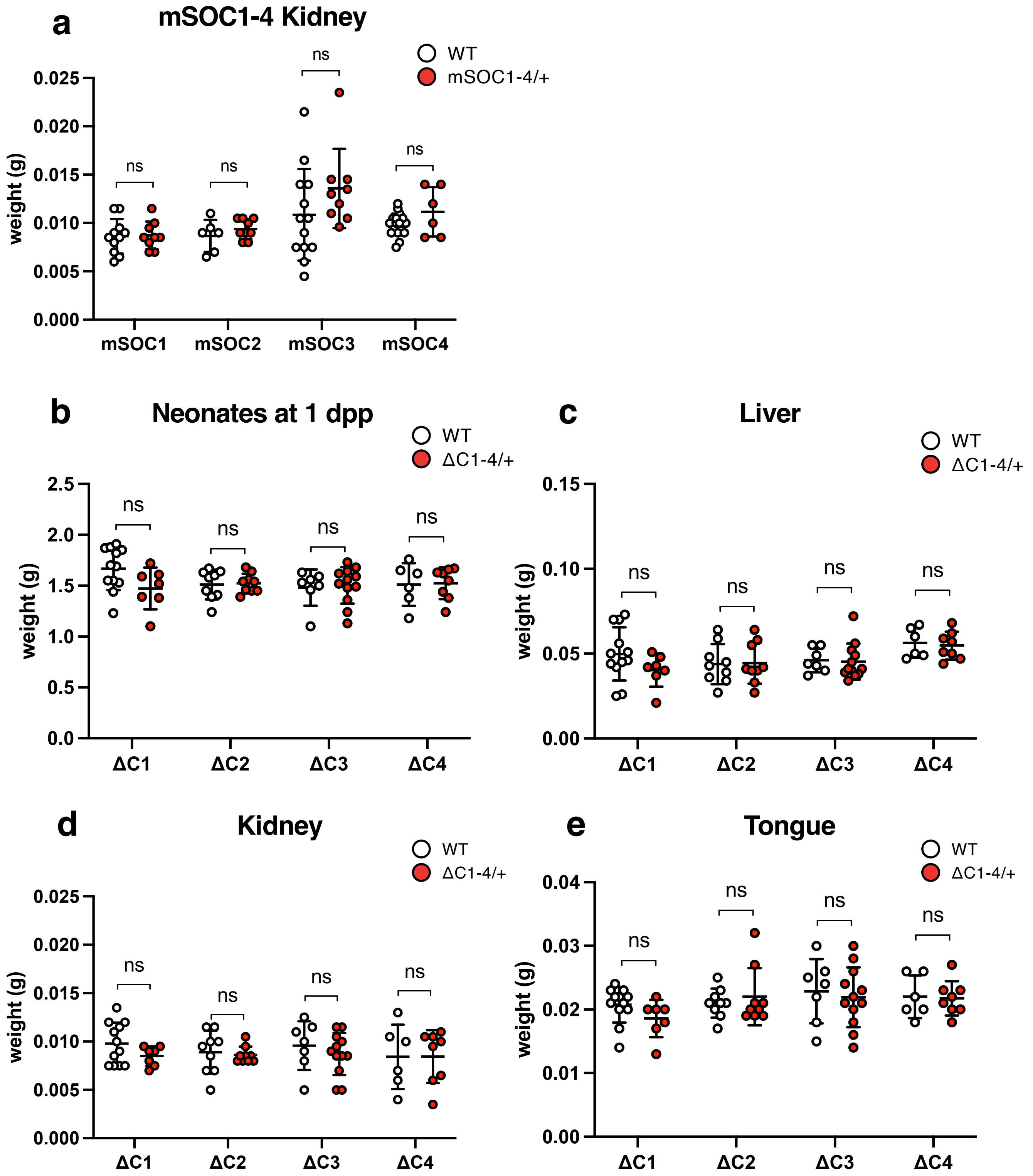

Supplementary Figure 6.

(a) Kidney weights at 1 dpp in *H19*-ICR$^{mSOC1/+}$ (WT and *H19*-ICR$^{mSOC1/+}$; n = 11 and n = 9, respectively, from 3 litters), *H19*-ICR$^{mSOC2/+}$ (WT and *H19*-ICR$^{mSOC2/+}$; n = 6 and n = 9, respectively, from 2 litters), *H19*-ICR$^{mSOC3/+}$ (WT and *H19*-ICR$^{mSOC3/+}$; n = 9 and n = 13, respectively, from 3 litters), and *H19*-ICR$^{mSOC4/+}$ (WT and *H19*-ICR$^{mSOC4/+}$; n = 19 and n = 6, respectively, from 3 litters). (b) Body weights at 1 dpp in *H19*-ICR$^{\Delta C1/+}$ (WT and *H19*-ICR$^{\Delta C1/+}$; n = 12 and n = 7, respectively, from 2 litters), *H19*-ICR$^{\Delta C2/+}$ (WT and *H19*-ICR$^{\Delta C2/+}$; n = 6 and n = 8, respectively, from 2 litters), *H19*-ICR$^{\Delta C3/+}$ (WT and *H19*-ICR$^{\Delta C3/+}$; n = 7 and n = 12, respectively, from 3 litters), and *H19*-ICR$^{\Delta C4/+}$ (WT and *H19*-ICR$^{\Delta C4/+}$; n = 6 and n = 8, respectively, from 2 litters). (c–e) Liver (c), tongue (d), and kidney weights (e) at 1 dpp in *H19*-ICR$^{\Delta C1/+}$ (WT and *H19*-ICR$^{\Delta C1/+}$; n = 12 and n = 7, respectively, from 2 litters), *H19*-ICR$^{\Delta C2/+}$ (WT and *H19*-ICR$^{\Delta C2/+}$; n = 6 and n = 8, respectively, from 2 litters), *H19*-ICR$^{\Delta C3/+}$ (WT and *H19*-ICR$^{\Delta C3/+}$; n = 7 and n = 12, respectively, from 3 litters), and *H19*-ICR$^{\Delta C4/+}$ (WT and *H19*-ICR$^{\Delta C4/+}$; n = 6 and n = 8, respectively, from 2 litters). White and red dots indicate individual weights of WT and heterozygous mutants with maternally transmitted *H19*-ICR$^{mSOC1-4}$ or *H19*-ICR$^{\Delta C1-4}$ alleles, respectively. Horizontal and error bars indicate the mean $\pm$ SD, respectively. ns; not significant (unpaired two-tailed *t*-test).

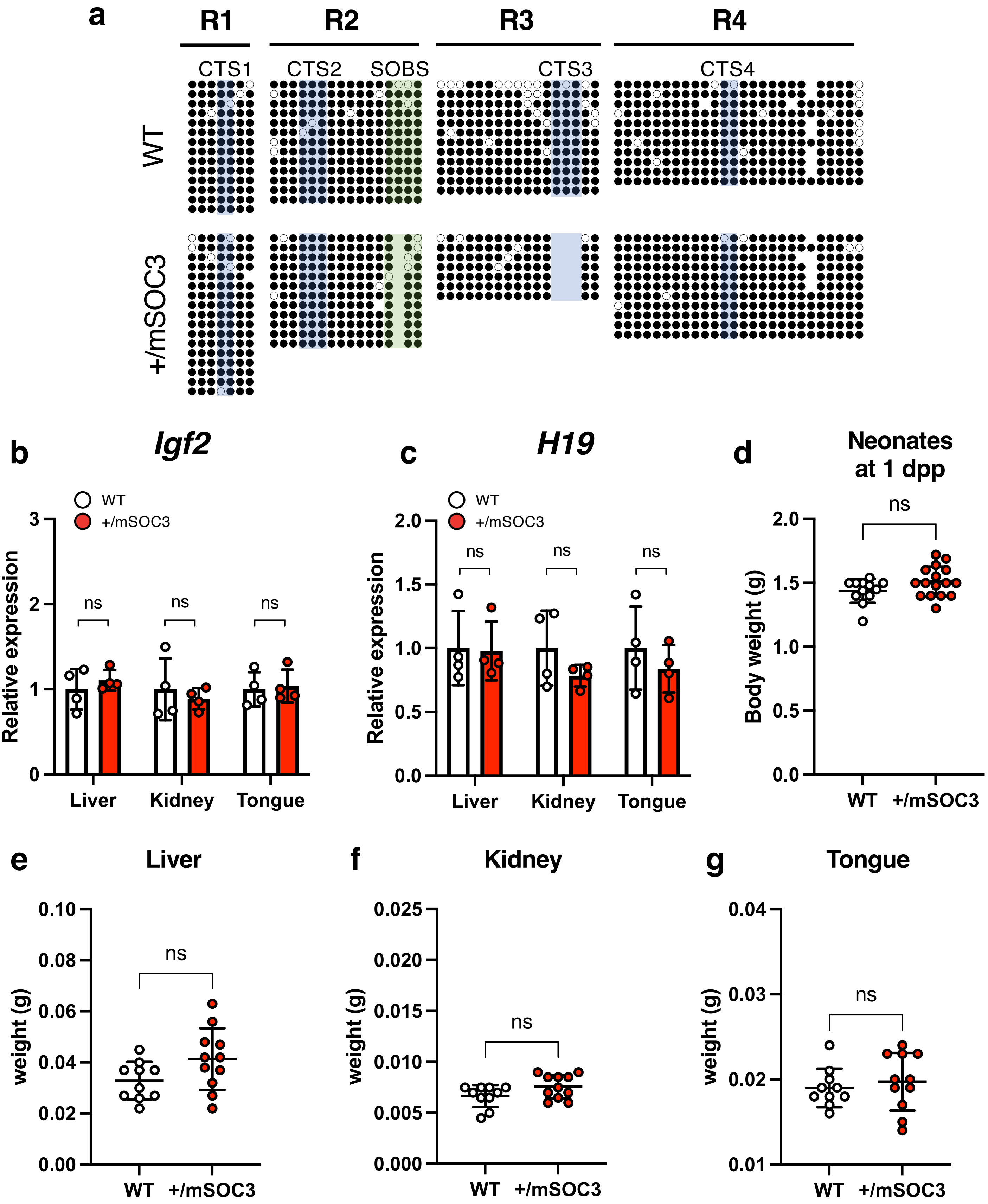

Supplementary Figure 7.
(a) Representative DNA methylation status at paternal *H19*-ICR in WT and *H19*-ICR$^{+/mSOC3}$ neonates. (b and c) Expression of *Igf2* (b) and *H19* (c) in WT (n = 4) and *H19*-ICR$^{+/mSOC3}$ (n = 4) tissues. Samples were collected from 2 litters. (d) Body weights at 1 dpp in *H19*-ICR$^{+/mSOC3}$ (WT and *H19*-ICR$^{\Delta C1/+}$; n = 12 and n = 16, respectively, from 3 litters. (e–g) Liver (e), kidney (f), and tongue weights (g) at 1 dpp in *H19*-ICR$^{+/mSOC3}$ (WT and *H19*-ICR$^{+/mSOC3}$; n = 10 and n = 11, respectively, from 3 litters). White and red dots indicate individual weights of WT and heterozygous mutants with paternally transmitted *H19*-ICR^{mSOC3} allele, respectively. Horizontal and error bars indicate the mean ± SD, respectively. ns; not significant (unpaired two-tailed *t*-test).

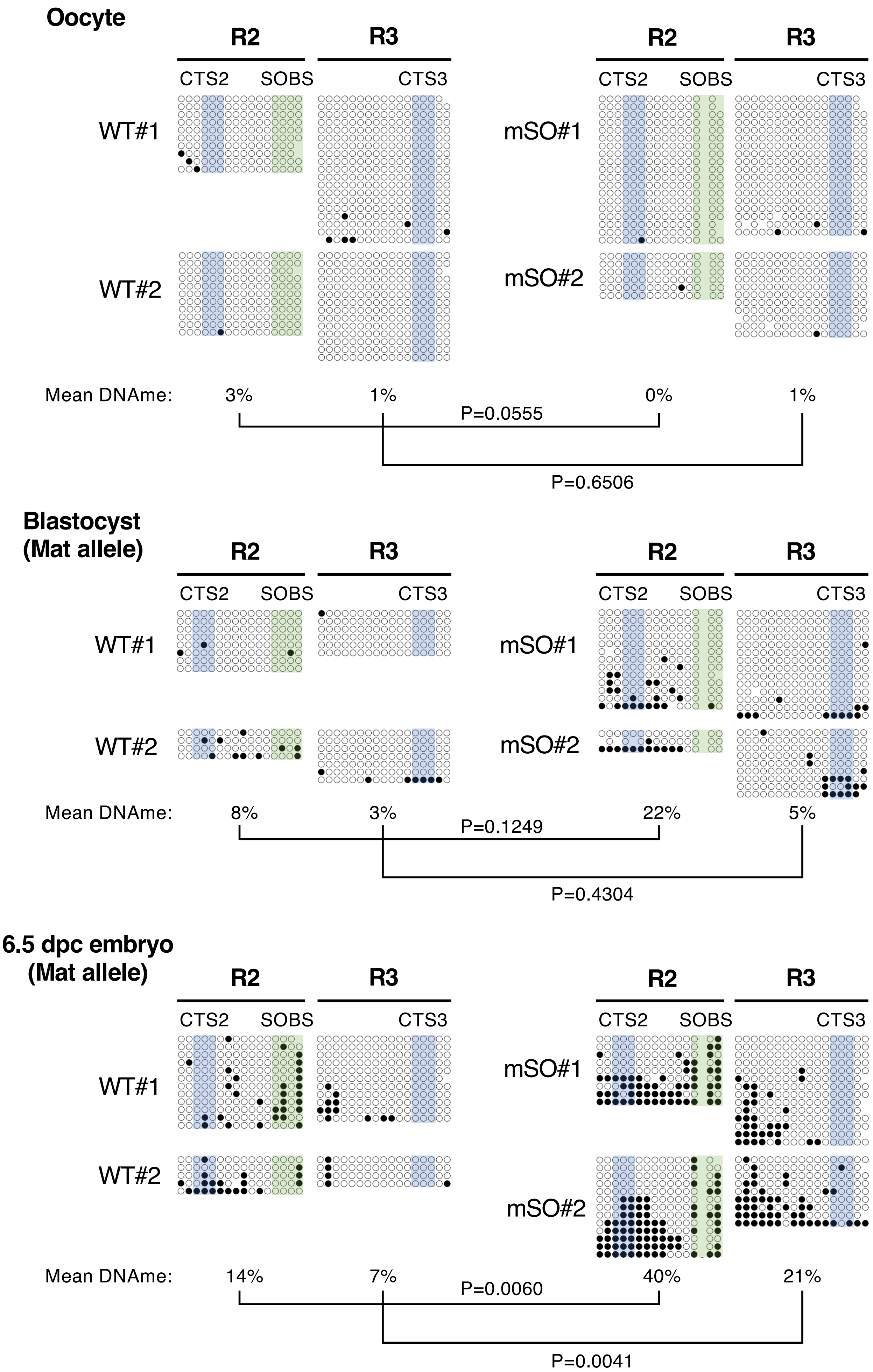

Supplementary Figure 8.
DNA methylation status of maternal *H19*-ICR in oocytes, blastocysts at 3.5 dpc, and embryos at 6.5 dpc. Methylation status in WT and *H19*-ICR$^{mSO/+}$ are shown. Percentages indicate mean DNA methylation levels of two replicates. *P*-values show statistical differences between WT and *H19*-ICR$^{\Delta C1\text{-}4/+}$ (Mann-Whiteney U test).

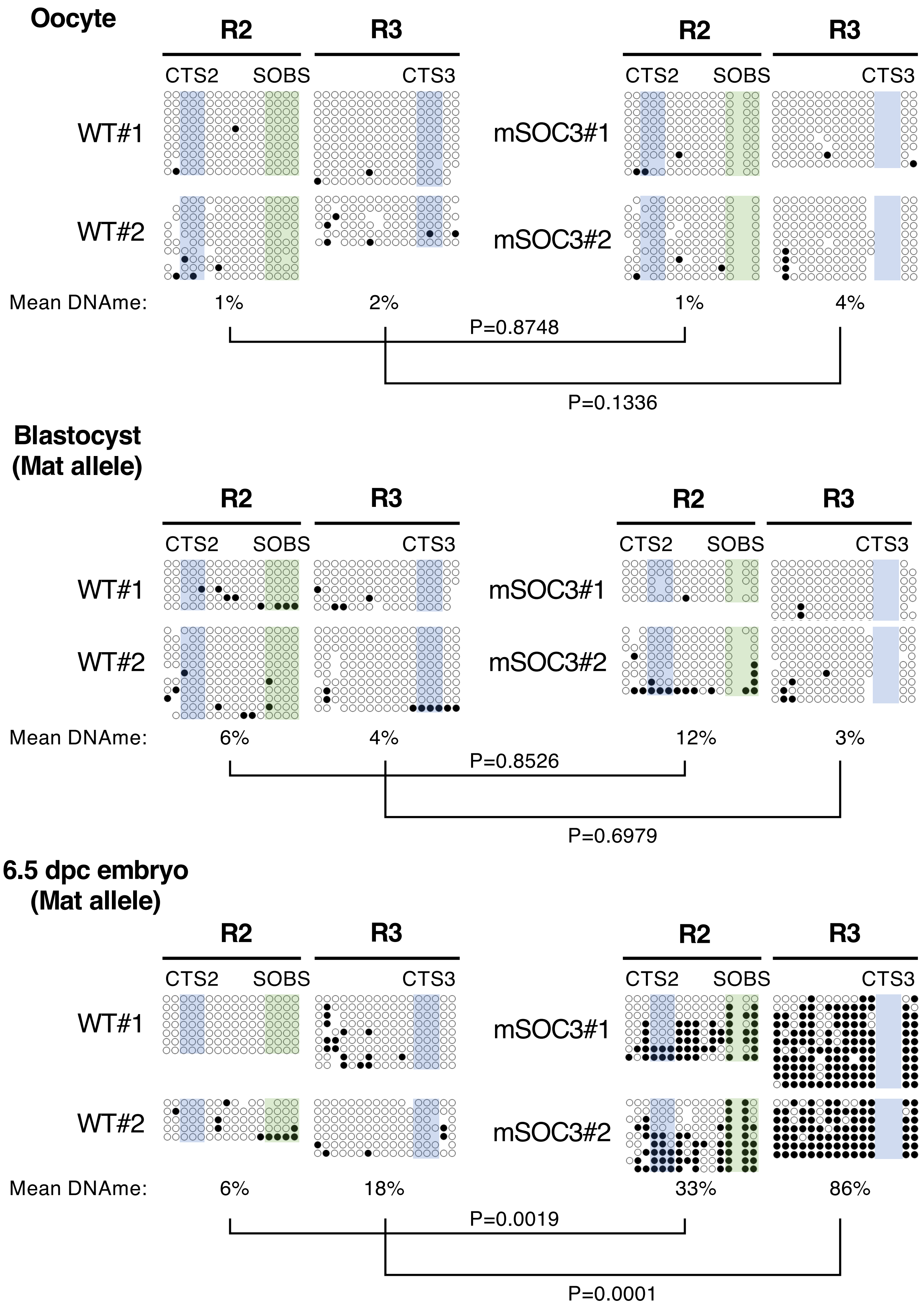

Supplementary Figure 9.
DNA methylation status of maternal *H19*-ICR in oocytes, blastocysts at 3.5 dpc, and embryos at 6.5 dpc. Methylation status in WT and *H19*-ICR$^{mSOC3/+}$ are shown. Percentages indicate mean DNA methylation levels of two replicates. *P*-values show statistical differences between WT and *H19*-ICR$^{\Delta C1\text{-}4/+}$ (Mann-Whiteney U test).

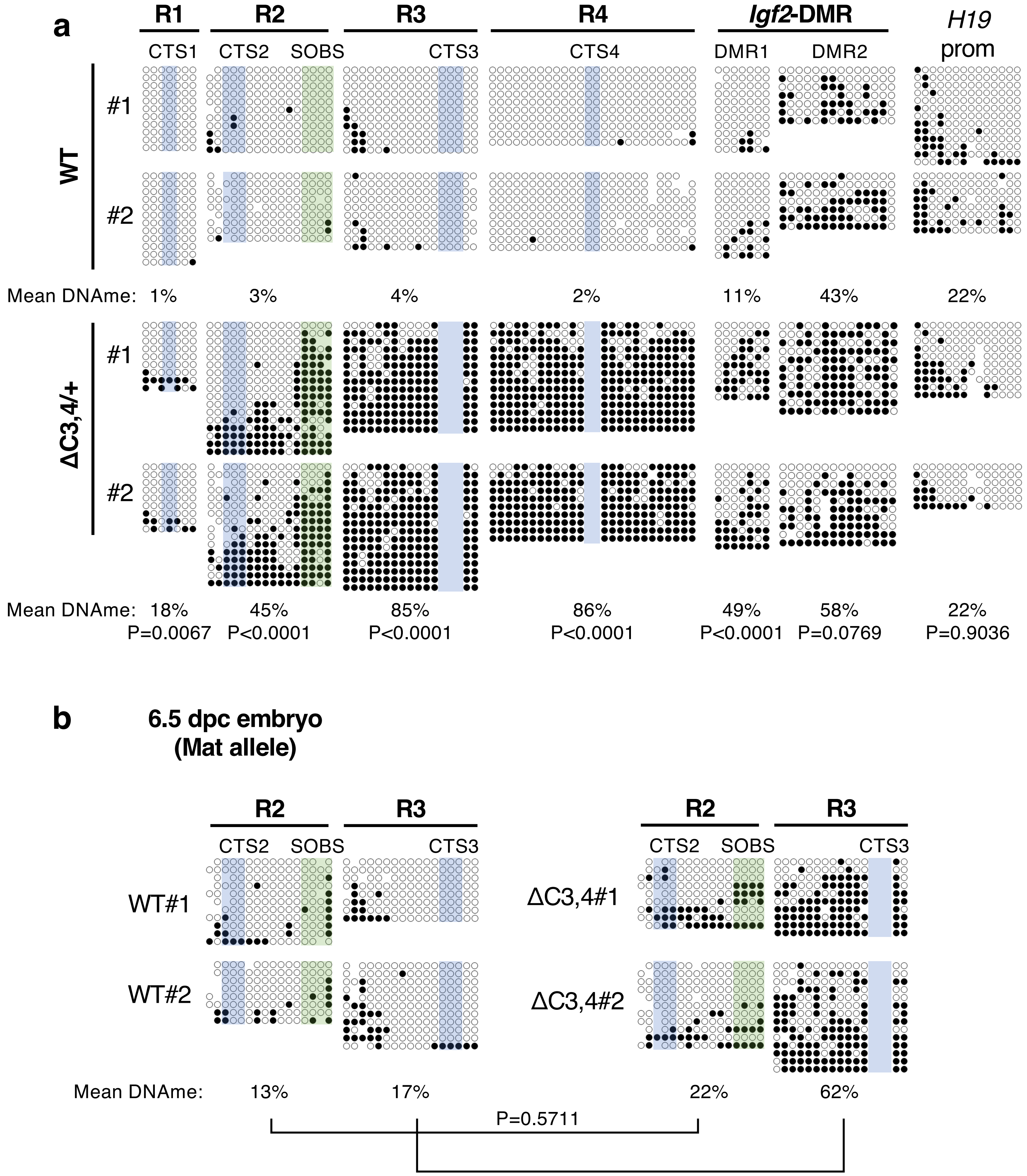

Supplementary Figure 10.

(a) Full results of the methylation status of the maternal allele in WT and *H19*-ICR$^{\Delta C3,4/+}$ neonates. (b) DNA methylation status of maternal *H19*-ICR in *H19*-ICR$^{\Delta C3,4/+}$ embryos at 6.5 dpc. Percentages indicate mean DNA methylation levels of two replicates. *P*-values show statistical differences between WT and *H19*-ICR$^{\Delta C3,4/+}$ embryos (Mann-Whiteney U test).

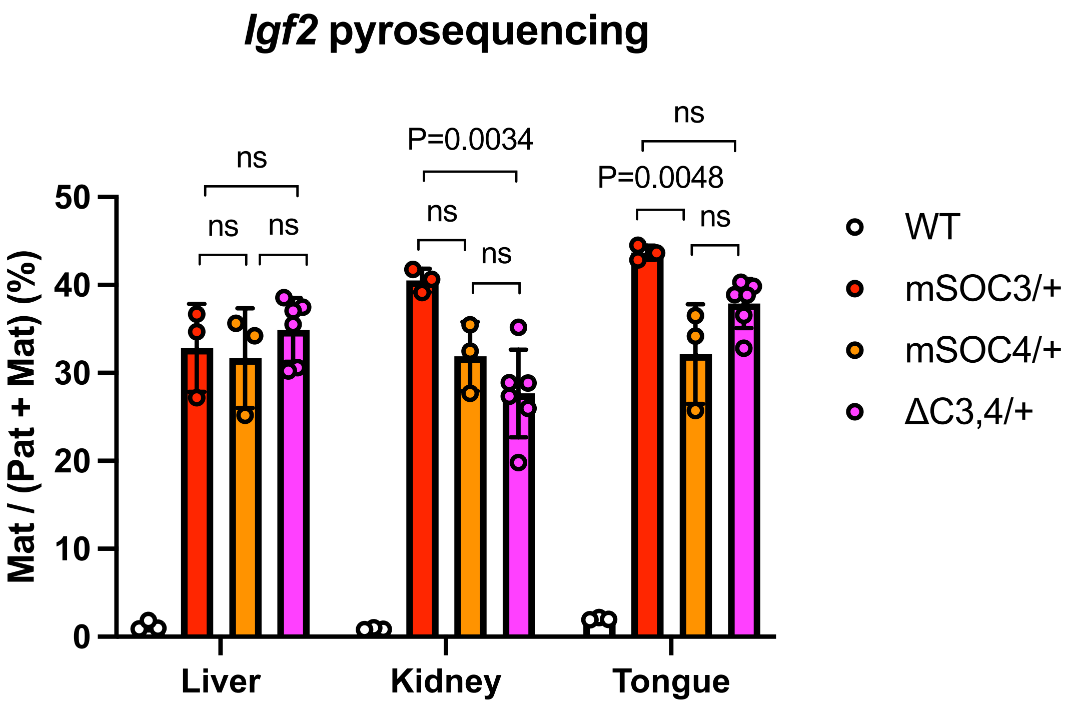

Supplementary Figure 11.
Quantitative allelic expression analysis of *Igf2* in WT (n = 3), *H19*-ICR$^{mSOC3/+}$ (n = 3), *H19*-ICR$^{mSOC4/+}$ (n = 3), and *H19*-ICR$^{\Delta C3,4/+}$ (n = 6) tissues. All samples were collected from 2 litters. All results are shown as the mean ± SD. Statistical analysis using one-way ANOVA are shown. Liver: $F\ (3, 11) = 51.56$, Kidney; $F\ (3, 11) = 61.19$, Tongue; $F\ (3, 11) = 114.9$. *P*-values are indicated.

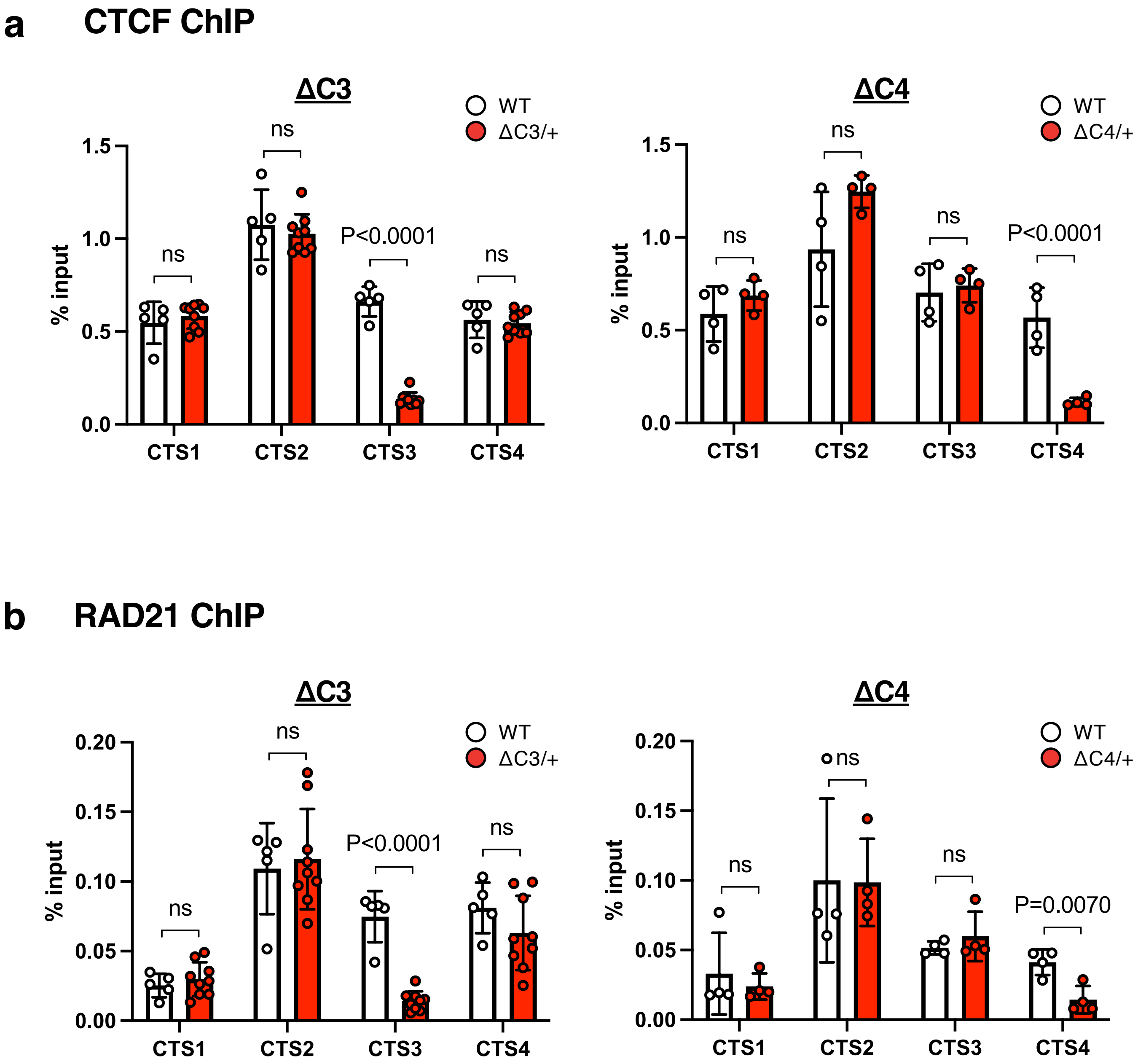

Supplementary Figure 12.
ChIP-qPCR analysis of CTS1-4 in *H19*-ICR$^{\Delta C3/+}$ (n = 9) and WT (n = 5), as well as *H19*-ICR$^{\Delta C4/+}$ (n = 4) and WT (n = 4) embryos from 2 litters using CTCF (a) and RAD21 (b) antibodies. White and red bars indicate WT and mutant, respectively. Error bars indicate standard deviation. *P*-values are indicated (unpaired two-tailed *t*-test). ns; not significant.

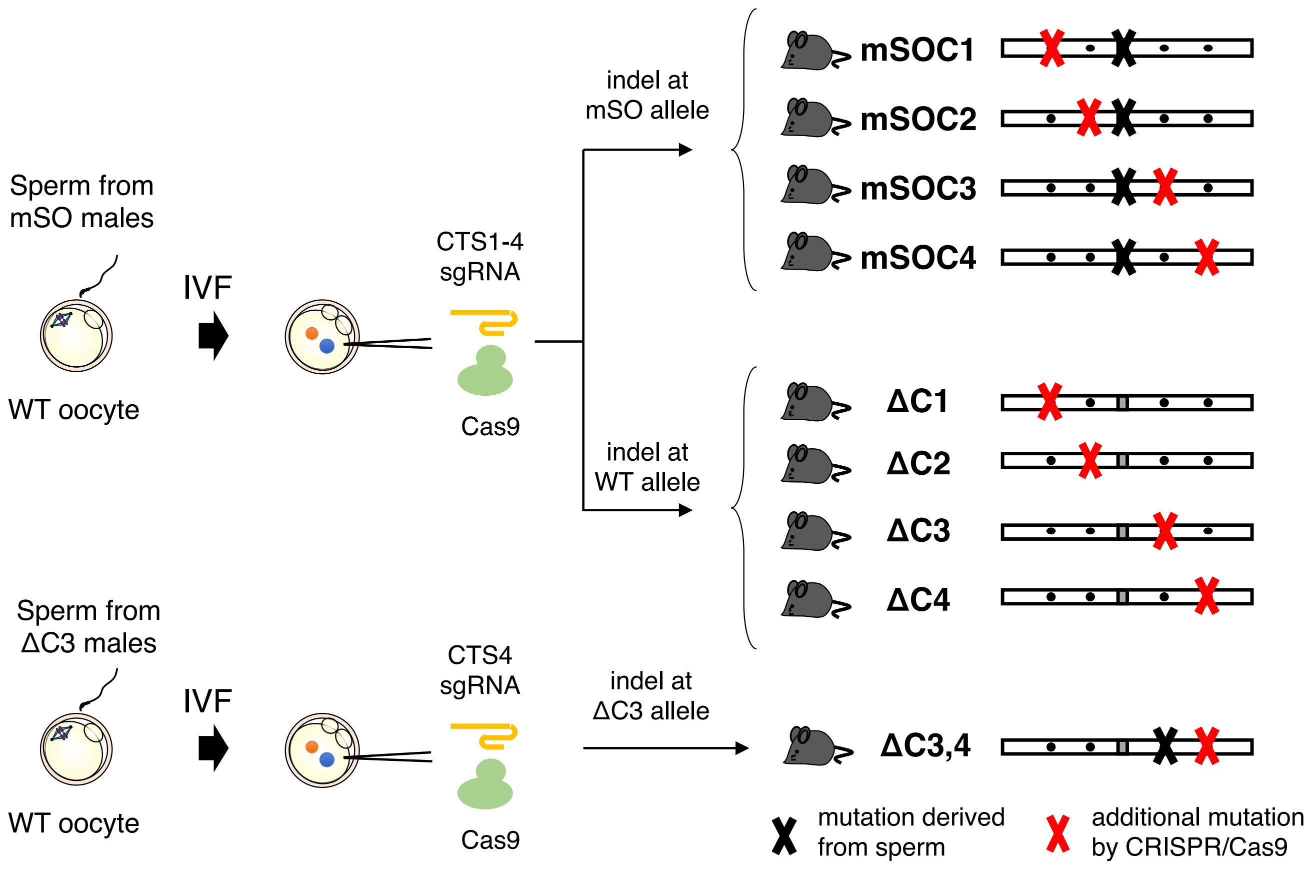

Supplementary Figure 13.
Schematic representation of the generation of *H19*-ICR$^{mSOC1\text{-}4}$, *H19*-ICR$^{\Delta C1\text{-}4}$, and *H19*-ICR$^{\Delta C3,4}$ mice.

A

```
                                CTS1
WT     GGGTTTATACGCGGGAGTTGCCGCGTGGTGGCAGCAAAATCGAT
mSOC1 GGGTTTATACGCGGGA-----------GTGGCAGCAAAATCGAT
                             CTS2
WT     CGATGTACGAGACTTCACTGCCGCCGTGCGGCAACCCTGGTCTTT
mSOC2 CGATGTACGAGACTTCACTGC--------GGCAACCCTGGTCTTT
                 CTS3
WT     GGAGTATGCTGCCACCGCGCGGTAGCATCCGTTCCCTTGTTGCACA
mSOC3 GGAGTATGCTatat------------ATCCGTTCCCTTGTTGCACA
                               CTS4
WT     GCAATATGTAGTATTGTACTGCCACCACGCGGCATCGTCTGTCCAT
mSOC4 GCAATATGTAGTATTGTACTG-----------CATCGTCTGTCCAT
```

B

```
                                CTS1
WT     GGGTTTATACGCGGGAGTTGCCGCGTGGTGGCAGCAAAATCGAT
ΔC1    GGGTTTATACGCGGGAGTTGC-----------AGCAAAATCGAT
                             CTS2
WT     CGATGTACGAGACTTCACTGCCGCCGTGCGGCAACCCTGGTCTTT
ΔC2    CGATGTACGAGACTTCACTGCCG--------CAACCCTGGTCTTT
                 CTS3
WT     GGAGTATGCTGCCACCGCGCGGTAGCATCCGTTCCCTTGTTGCACA
ΔC3    GGAGT---------------------ATCCGTTCCCTTGTTGCACA
                               CTS4
WT     GCAATATGTAGTATTGTACTGCCACCACGCGGCATCGTCTGTCCAT
ΔC4    GCAATATGTAGTATTGTACTGtacag---CGGCATCGTCTGTCCAT
```

C

```
                 CTS3
WT     GGAGTATGCTGCCACCGCGCGGTAGCATCCGTTCCCTTGTTGCACA
ΔC3,4 GGAGT---------------------ATCCGTTCCCTTGTTGCACA
                              CTS4
WT     GCAATATGTAGTATTGTACTGCCACCACGCGGCATCGTCTGTCCAT
ΔC3,4 GCAATATGTAGTATTGTACTGC-----------ATCGTCTGTCCAT
```

Supplementary Figure 14.
Genomic sequences of *H19*-ICR$^{mSOC1\text{-}4}$, *H19*-ICR$^{\Delta C1\text{-}4}$, and *H19*-ICR$^{\Delta C3,4}$. Nucleotide sequences of SOBS. WT and mutant alleles are shown. Each CTS is highlighted. Each target sequence of sgRNA is underlined. Deleted nucleotides are shown with hyphens. An unexpected insertion is indicated with small letters.

Supplementary Table 1. ssODN and primers for sgRNA template.

Primer	Sequence (5’→3’)
mSO	TTGATGTAGGATTCCTCAGCTGCCAAGCTGGCAGCTGAGCCCCATTGAGAGAGAATGCAGctcagcttcTtaccGgtatgacactaCactagTCATgattATCTtcgttCCCCAATgAttATACctagGacgtAgcgcttAGACTTTCTTGTTGGCGGTTCCTTAAGTGATTCTTTGGGTAGGGAGGTCAGG
sgSO_F	CTAATACGACTCACTATAGgcgctttcgtattctaattgGTTTTAGAGCTAGAAATAGCA
sgCTS1_F	CTAATACGACTCACTATAGtcgattttgctgccaccacgGTTTTAGAGCTAGAAATAGCA
sgCTS2_F	CTAATACGACTCACTATAGgagacttcactgccgccgtgGTTTTAGAGCTAGAAATAGCA
sgCTS3_F	CTAATACGACTCACTATAGgaacggatgctaccgcgcggGTTTTAGAGCTAGAAATAGCA
sgCTS4_F	CTAATACGACTCACTATAGggacagacgatgccgcgtggGTTTTAGAGCTAGAAATAGCA

Supplementary Table 2. Genotyping primers.

Primer	Sequence (5’→3’)
H19_SOM_geno_F1	TAGGTGTCCTGCCTTCTGCT
H19_SOM_geno_F2	CAAAACAGGTGAACCCCAAC
H19_C3C4_geno_F1	AAGGGGACCATTCCGTAAGT
H19_C3C4_geno_R1	GCTATGCCTCAGTGGTCGAT
CTCF2_geno_F1	TGTGCAGAATTTGAGGACCA
CTCF2_geno_R1	GATTGCGCCAAACCTAAAGA
CTCF1_geno_R1	AGTTCCCGAATCACCACAAG

Supplementary Table 3. Primers for allelic expression analysis.

Primer	Sequence (5’→3’)
Igf2_3UTR_allele_F	AGGAGTGGGCAAGATGACAC
Igf2_3UTR_allele_R	TCTATCGCTTGGCCTGAGTT
H19_allele_F	CAGACTAGGCGAGGGGAAG
H19_allele_R	CCATCTTCATGGCCAACTCT
Igf2ex6_pyro_F1	CGACGCGTGGGAAGTCTT
Igf2ex6_pyro_R1_Bio	[5’Biotin]-TGGCCTCCTATGCCTAGTGTG
Igf2ex6_pyro_seq	ACAAAGGGAGGGACC

Supplementary Table 4. Primers for bisulfite sequencing.

Primer	Sequence (5’→3’)
H19_C1as_BSF1	AAGGAGATTATGTTTTATTTTTGGA
H19_C1as_BSR1	CCCACAACATTACCATTTATAAATTC
H19_C2SOas_BSF1	GGGGGGTTATAAATGTTATTAGGGGGG
H19_C2SOas_BSF3 (nested)	GGGGGGTAGGATATATGTATTTTTTAGGTTGG
H19_C2SOas_BSR1	CTACCAAACTAACAACTAAACCCC
H19_C3as_BSF1	GGGGTTTAGTTGTTAGTTTGGTAGTTGAGG
H19_C3as_BSF2 (nested)	GAAGAGGTGGTAGGATTTTTGGTTGG
H19_C3as_BSR1	CACACATCTTACCACCCCTATAAATCCC
H19_C4_BSF3	GGTTATTTGGGATATTGTAATGGTTG
H19_C4_BSR2	CACCTCTAAAATAATTCCCTTACACAC
Igf2-DMR1_7CpG_BSF1	GAATGTTTGTAGGAGTTTTAGGGAG
Igf2-DMR1_7CpG_BSR1	CCCCCAAATTTACTTCTAACTCCTAACC
Igf2-DMR2_BSF1	GGGTTGGGGTGGTTATTTTAATGGGTG
Igf2-DMR2_BSR1	CACCCCACCTCAAAACAACTTCCC
H19prom_BSF1	GATAGGGTTTGGTATTGTTTTTAG
H19prom_BSR1	CACTTCCATCTAAAACTACTCTACTCTC

Supplementary Table 5. Primers for ChIP-qPCR.

Primer	Sequence (5’→3’)
H19_CTCF1_ChIP_F1	CTCTTTAGGTTTGGCGCAAT
H19_CTCF1_ChIP_R1	GAATCAGTTGTGGGGTTTATACG
H19_CTCF2_ChIP_F2	GCCCATGACTATGGGATCAT
H19_CTCF2_ChIP_R1	GACTCGGACTCCCAAATCAA
H19_CTCF3_ChIP_F2	GGGATATTGCTGGGAATGAA
H19_CTCF3_ChIP_R2	TTATGTGCAACAAGGGAACG
H19_CTCF4_ChIP_F2	CGTCTGCCGAGCAATATGTA
H19_CTCF4_ChIP_R2	TCGATCGGTTCACTCTCCAC
