# Supplementary material for: Identification of responsible sequences which mutations cause maternal H19-ICR hypermethylation with Beckwith–Wiedemann syndrome-like overgrowth
Source: Commun Biol. 2024 Dec 2;7:1605. doi: 10.1038/s42003-024-07323-x (PMC11612015; doi:10.1038/s42003-024-07323-x)
Supplement: Supplementary file 3 — Description of Additional Supplementary Files [file 42003_2024_7323_MOESM3_ESM.pdf]

Description of Additional Supplementary Files

File name: Supplementary Data

Description: The numeral source data behind the graphs in the main and the supplementary figures.
