# Supplementary material for: Identification of responsible sequences which mutations cause maternal H19-ICR hypermethylation with Beckwith–Wiedemann syndrome-like overgrowth
Source: Commun Biol. 2024 Dec 2;7:1605. doi: 10.1038/s42003-024-07323-x (PMC11612015; doi:10.1038/s42003-024-07323-x)
Supplement: Supplementary file 5 — Reporting summary [file 42003_2024_7323_MOESM5_ESM.pdf]

nature portfolio

Corresponding author(s): Satoshi Hara
Last updated by author(s): Nov 21, 2024

Reporting Summary

Nature Portfolio wishes to improve the reproducibility of the work that we publish. This form provides structure for consistency and transparency in reporting. For further information on Nature Portfolio policies, see our Editorial Policies and the Editorial Policy Checklist.

Statistics

For all statistical analyses, confirm that the following items are present in the figure legend, table legend, main text, or Methods section.

n/a	Confirmed	
☐	☒	The exact sample size (*n*) for each experimental group/condition, given as a discrete number and unit of measurement
☐	☒	A statement on whether measurements were taken from distinct samples or whether the same sample was measured repeatedly
☐	☒	The statistical test(s) used AND whether they are one- or two-sided<br>*Only common tests should be described solely by name; describe more complex techniques in the Methods section.*
☒	☐	A description of all covariates tested
☒	☐	A description of any assumptions or corrections, such as tests of normality and adjustment for multiple comparisons
☐	☒	A full description of the statistical parameters including central tendency (e.g. means) or other basic estimates (e.g. regression coefficient) AND variation (e.g. standard deviation) or associated estimates of uncertainty (e.g. confidence intervals)
☐	☒	For null hypothesis testing, the test statistic (e.g. F, t, r) with confidence intervals, effect sizes, degrees of freedom and P value noted<br>*Give P values as exact values whenever suitable.*
☒	☐	For Bayesian analysis, information on the choice of priors and Markov chain Monte Carlo settings
☒	☐	For hierarchical and complex designs, identification of the appropriate level for tests and full reporting of outcomes
☒	☐	Estimates of effect sizes (e.g. Cohen's d, Pearson's r), indicating how they were calculated

Our web collection on statistics for biologists contains articles on many of the points above.

Software and code

Policy information about availability of computer code

Data collection	Data was collected using QuantStudio Design & Analysis Software (qPCR) and PyroMark Q24 Software (pyrosequencing).
Data analysis	Quantification tool for Methylation Analysis (QUMA, http://quma.cdb.riken.jp/) was used for analyses of bisulfite sequencing data. Statistical analyses were performed using QUMA and GraphPad Prism 10.

For manuscripts utilizing custom algorithms or software that are central to the research but not yet described in published literature, software must be made available to editors and reviewers. We strongly encourage code deposition in a community repository (e.g. GitHub). See the Nature Portfolio guidelines for submitting code & software for further information.

Data

Policy information about availability of data

All manuscripts must include a data availability statement. This statement should provide the following information, where applicable:

- Accession codes, unique identifiers, or web links for publicly available datasets
- A description of any restrictions on data availability
- For clinical datasets or third party data, please ensure that the statement adheres to our policy

All of the data supporting the findings of this study are available in this article and in the Supplementary information.The data that support this study are available from the corresponding author upon reasonable request. Supplementary data (Source data) are provided with this paper.

Research involving human participants, their data, or biological material

Policy information about studies with human participants or human data. See also policy information about sex, gender (identity/presentation), and sexual orientation and race, ethnicity and racism.

Reporting on sex and gender	Not applicable.
Reporting on race, ethnicity, or other socially relevant groupings	Not applicable.
Population characteristics	Not applicable.
Recruitment	Not applicable.
Ethics oversight	Not applicable.

Note that full information on the approval of the study protocol must also be provided in the manuscript.

Field-specific reporting

Please select the one below that is the best fit for your research. If you are not sure, read the appropriate sections before making your selection.

☒ Life sciences ☐ Behavioural & social sciences ☐ Ecological, evolutionary & environmental sciences

For a reference copy of the document with all sections, see nature.com/documents/nr-reporting-summary-flat.pdf

Life sciences study design

All studies must disclose on these points even when the disclosure is negative.

Sample size	Sample sizes were determined based on published studies in the field or our previous experiences. No statistics was used to predetermine the sample size.
Data exclusions	No data were excluded from analyses.
Replication	Experiments were performed using at least two biological replicates.
Randomization	Wild-type and mutant mice used for analysis were chosen randomly within each group.
Blinding	The investigators were not blinded for group allocation due to feasibility of experiments.

Reporting for specific materials, systems and methods

We require information from authors about some types of materials, experimental systems and methods used in many studies. Here, indicate whether each material, system or method listed is relevant to your study. If you are not sure if a list item applies to your research, read the appropriate section before selecting a response.

Materials & experimental systems

n/a	Involved in the study	
☐	☒	Antibodies
☒	☐	Eukaryotic cell lines
☒	☐	Palaeontology and archaeology
☐	☒	Animals and other organisms
☒	☐	Clinical data
☒	☐	Dual use research of concern
☒	☐	Plants

Methods

n/a	Involved in the study	
☒	☐	ChIP-seq
☒	☐	Flow cytometry
☒	☐	MRI-based neuroimaging

Antibodies

Antibodies used	anti-CTCF (#3418; Cell Signaling Technology) and anti-Rad21 (ab992; Abcam) were used for ChIP-qPCR experiments.

Validation	All primary antibodies used in this study have been validated in mice according to the manufacturer's instruction and previous studies.

Animals and other research organisms

Policy information about studies involving animals; ARRIVE guidelines recommended for reporting animal research, and Sex and Gender in Research

Laboratory animals	C57BL/6 background wild-type or mutated mice and PWK/PhJ mice, including both males and females, were used for experiment at 6.5 dpc, 10.5 dpc, 15.5 dpc, 16.5 dpc, or 1–21 dpp, as described in the Methods and Figure Legends.
Wild animals	No wild animals were used in this study.
Reporting on sex	Both male and female fetus or offspring were used in the experiments. Sex-based analyses were not conducted.
Field-collected samples	No field-collected samples were used in this study.
Ethics oversight	All animal protocols were approved by the Animal Care and Use Committee of Saga University, Saga, Japan. All experiments were conducted in accordance with the Regulations on Animal Experimentation of Saga University.

Note that full information on the approval of the study protocol must also be provided in the manuscript.

Plants

Seed stocks	Not applicable.
Novel plant genotypes	Not applicable.
Authentication	Not applicable.
